# Opportunities for extended community pharmacy services in United Arab Emirates: perception, practice, perceived barriers and willingness among community pharmacists

**DOI:** 10.1186/s40545-022-00418-y

**Published:** 2022-03-23

**Authors:** Subish Palaian, Muaed Alomar, Nageeb Hassan, Fatima Boura

**Affiliations:** Department of Clinical Sciences, College of Pharmacy and Health Sciences, Ajman University, Ajman, United Arab Emirates

**Keywords:** Community pharmacy services, Extended community pharmacy services, Perception, United Arab Emirates

## Abstract

**Background:**

Community pharmacies are widely distributed in the United Arab Emirates where community pharmacists’ (CPs’) perspectives on provision of extended community pharmacy services (ECPS) is not known. This study assessed CPs perception, practices, perceived barriers and willingness to provide ECPS.

**Methods:**

A descriptive cross-sectional survey using a self-administered Likert-type questionnaire (Cronbach alpha = 0.976) was conducted among 195 community CPs in Northern emirates, selected through multistage sampling technique. The filled questionnaires were assessed for CPs’ perception, practice, perceived barriers and willingness to perform ECPS. Mann–Whitney and Kruskal–Wallis tests examined the association between demography and outcome variables (alpha value of 0.05) with further analysis using Dunn’ post hoc test.

**Results:**

Of the respondents, 71.8% (*n* = 140) ‘strongly agreed’ that every CP must provide ECPs and 39% (*n* = 76) ‘strongly agreed’ in supporting ECPS with another 44.1% (*n* = 86) ‘agreed’ on the same. The major barriers felt by CPs in practicing ECPS were lack of incentive for employee pharmacists [3 (2–3)], lack of documentation [3 (2–3)], insufficient time [3 (2–3)] and lack of patients’ demand [(2–3)]; maximum score (5–5). CPs also responded ‘completely’ willing to provide services such as blood pressure measurement, pregnancy testing and BMI measurement. Pharmacy location influenced practice score (*p* = 0.008) and access to the internet had a significant effect on barriers score (*p* = 0.000). Availability of drug information sources impacted perception (*p* = 0.038), practice (*p* = 0.000) and willingness scores (*p* = 0.011).

**Conclusions:**

CPs’ perception on providing ECPS was positive and they are currently offering ECPS within their available resources and facilities. Less demand from patients and lack of time were reportedly the major barriers in offering ECPS. Proper utilization of CPs in providing ECPS can complement the healthcare system with additional cost and time savings for the patients.

**Supplementary Information:**

The online version contains supplementary material available at 10.1186/s40545-022-00418-y.

## Background

Community pharmacists (CPs) are the most accessible healthcare professionals available to the public without an appointment and a consultation fee. Community pharmacies are often the first place most people visit to receive health information and medical support [1, 2]. CPs deals with and meeting the demands of different strata of society and hence are in a powerful position to handle people with mild symptoms requiring over-the-counter (OTC) medications in addition to chronically ill patients [2, 3]. The nature of pharmacy practice has changed recently with pharmacists work in close contact with patients [4–6]. There are many studies reporting usefulness of CPs in delivering patientcare in multiple ways such as identifying drug therapy-related problems, patient counseling and enhancing patients’ quality of life. Few examples of CP contributing to clinical services include diabetes [7, 8] and asthma [9, 10] with significant clinical outcomes have been achieved.

Extended community pharmacy services (ECPS) present the idea that CP's responsibilities are more than dispensing medicines [11]. Accordingly, CPs roles were extended to comprise additional patient-oriented services including; disease management, medicines use reviews, monitoring and screening of drug therapy, educating patients to improve drug adherence and lifestyle modifications, and improving patient counseling [12]. In addition to that, ECPS suggests that pharmacists should be on the front line to guarantee the safe use of drugs during pregnancy and lactation [13]. A detailed list on various ECPS provided by pharmacists globally are summarized by Nordin et al. [14].

The benefits of ECPS are numerous and it could eventually lead to several advantages to patients including; positive contribution to patient care, optimize drug therapy, a reduction in the workload of general practitioners, lowering the cost of long-term healthcare, and health improvement of the chronically ill patients [4, 15]. The potential advantages for pharmacists while providing ECPS involve enhancement of their professional qualifications and profession satisfaction [4]. However, the potential disadvantages of ECPS may be rising working load of pharmacists and developing a possible tense relationship between pharmacists and physicians [4].

In United Kingdom (UK), ECPS is practiced and pharmacist services are classified into essential (repeat dispensing of chronic medication prescriptions), enhanced (mild diseases prescribing), and advanced (identify ineffective use of medications. identify side effects and drug interactions, and cost-effectiveness of the medications) [4, 16]. Also, pharmacists in the UK are permitted to dispense some prescribing only medicines without a prescription under specific regulations [17]. In the United States (USA), wherein services like administering vaccines, prescribing medications for some chronic diseases, laboratory testing and creating care plans are now practiced by CPs [15]. Moreover, pharmacists in the USA may apply for additional certification to become specialized [15]. These scenarios clearly depict the potential for CPs to perform ECPS which can eventually offer multiple advantages to the patients, healthcare system and to the pharmacists themselves in terms of professional satisfaction and better business volume.

A closer look at the community pharmacy practice in developing countries inadvertently reveals the need for reframing the practice standards [1]. Similarly, in UAE and several other Arab countries with nearly the same community pharmacy practice scenario, ECPS are either minimally practiced or not actually practiced yet.like: Some barriers for the lack of practice includes; pharmacists’ attitudes (fear of change), lack of advanced practice qualifications (communications), resource limitations (finance), system limitations (physician/nurse resistance), intraprofessional obstacles, and educational berries [4, 18, 19]. In a customer perception survey carried out in the UAE, the perceptions of patients regarding CPs were unsatisfied and not appreciable [20]. Findings have shown that the majority of pharmacists in the UAE give information regarding dosage and frequency of drug use only; they rarely inform the patient about possible ADR, except if requested by the patients [4, 21].

UAE has recently adopted various steps to introduce the concept of ECPS [22]. In order to accomplish the quality of healthcare, the health authority of Abu Dhabi (HAAD) added extended pharmacy services to its strategy to meet the current needs. It established many programs to train CPc to apply ECPS [4]. The UAE has approximately 1300 licensed community pharmacists work in the private sector [22, 23] Few pharmacies open 24 h a day [4] and most of them are open with an average working day of 13 h [23].

According to the latest United Nations data 2020, the estimated population of the UAE is around 10 million, which is 0.13% of the entire world population [24]. In 2011, the average per capita expenditure of the healthcare in UAE was around 1500 $USA [25]. Therefore, implementing ECPS may contribute to reduction of financial burden on the society, as it is estimated that cost-effectiveness can be achieved with ECPS provision [26]. Currently, there is a lack of comprehensive research on ECPS in UAE. Introducing ECPS requires improvements at multiple levels including; pharmacy education, regulatory changes, licensing requirements for pharmacists, etc. It is also crucial to know the perception, practices, perceived barriers and willingness of currently practicing CPs toward ECPS. Understanding these aspects can help in initiating appropriate strategies while implementing these services and help pharmacy educators, policy-makers and practitioners. Therefore, this research attempted to assess UAE CPs' perception, practices, perceived barriers and willingness toward the provision of ECPS.

## Methods

### Study design

A descriptive, cross-sectional self-administered survey was conducted among CPs in northern emirates (Sharjah, Ajman and Um-al-Quwain) of the UAE. The healthcare in these three emirates is governed by the Ministry of health (MOH).

### Study duration

Data collection was conducted during 15th February 15th April, 2020. Since then due to the implementation of COVID-19 lockdown in UAE the data collection had to be stopped.

### Inclusion and exclusion criteria

All CPs in northern emirates registered with the MOH and working during the business hours from both genders were included in the sampling frame. However, the exclusion criteria were pharmacy technician, or trainee students, or CPs not willing to take part in the study.

### Sample size calculation

Sample size of the study was considered by referring to similar studies [27, 28], and calculated using Raosoft online sample size calculator as 260. The number of community pharmacies in Sharjah, Ajman and Umm Al Quwain were UAE is 802 [29, 30]. According to convenience, authors managed to collect responses from only 195 CPs. Further data collection could not be done due to the COVID-19 pandemic.

### Sampling method

This study utilized multistage sampling technique. This sampling was selected as it was the most proper data collection in the present study setting. The diversity of healthcare systems was considered while conducting this research. So, in the first stage, only northern emirates (Sharjah, Ajman and Umm Al Quwain), were selected by convenience sampling.

In the second stage, simple random sampling applied for the selection of the CPs by giving numbers to all community pharmacies in the northern emirates mentioned in the MOH list, and these numbers are placed and chosen randomly to ensure randomization. Only one pharmacist was nominated from each pharmacy to reach 195 responses. The selection was done at the same time of the visit by contacting the available licensed CPs.

### Research tools

#### Structured questionnaire development

The research team developed the questionnaire by referring to previous literature, and after considering the parameters to be assessed in the study [17, 27, 28, 31–33]. The 10-min questionnaire was designed to be self-administrated which contained five parts. Part I consisted of 17 questions covered the pharmacist and pharmacy-related information. Part II included 11 questions to assess the perception of the respondents toward ECPS., which includes 5 Likert items exploring the pharmacists’ perceptions, Part III consisted of 13 questions and includes domain related to practices, that consist of 5 Likert items, Part IV consisted of 26 items representing the barriers of reporting process and Part V assessed the CP’s willingness to provide six specific ECPS and an open-ended question requested the respondents to recommend any other services they provided in their pharmacies (see Additional file 1).

#### Scoring of the responses

Part I was about demography. Part II questions had 5 responses; strongly agree, agree, neutral, disagree, and strongly disagree. To establish the association between demographic variables and perception, practices, perceived barriers and willingness scores. The responses were scored as strongly agree 1, agree 2, neutral 3, disagree 4 and strongly disagree 5. Part III of the questionnaire had never, rare, occasionally, often, always and scores, 1, 2, 3, 4 and 5, respectively. Part IV items were expressed as a sequence of statements and the CPs were asked to specify their agreement or disagreement on a 5-point Likert scale from ‘strongly agree’ to ‘strongly disagree’. A score of 1, 2, 3, 4, 5 was given, respectively, for strongly agree, agree, neutral, disagree, and strongly disagree. Part V had three responses never, partly and completely and were scored 1, 2 and 3, respectively.

#### Validity of the study tool

The content validity of the tool was assessed based on the viewpoints of two experts in the related field [34]. One of them was a pharmacy manager in a chain pharmacy in UAE and another board certified pharmacotherapy specialist and lecturer at UAE university. Based on their input, the tool was modified and a draft version for pilot testing was prepared. Ten participants included in the pilot study to test the feasibility of data collection process, reliability of the questionnaire and measure the time needed for filling the questionnaire. The Cronbach alpha of the final version of the tool was 0.976 with no changes needed.

### Data collection

A Master in Clinical Pharmacy student (coauthor of this research) distributed and collected the questionnaire from the respondents. The data collecting pharmacist was trained to collect the questionnaires in an accumulative session for two hours to ensure the understanding of the questionnaire. She visited each pharmacy and invited the licensed pharmacists to participate. The study information sheet was distributed to CPs in addition to the consent form to be signed. The questionnaires were filled by the participants themselves and in some cases with few clarifications were sought from the data collector.

### Data analysis

Data from the survey were coded and entered into IBM Version SPSS 26. The distribution pattern of the data was assessed using Shapiro–Wilk at alpha = 0.05. The data were not normally distributed, as the *p* values were less than 0.01 using Shapiro–Wilk test. Mann–Whitney test and Kruskal–Wallis test were applied to assess the effect of sociodemographic data on the study outcomes. The *p* value of < 0.05 was considered as statistically significant. Post hoc Dunn test was applied further to the statistically significant values as applicable.

## Results

### Pharmacists’ sociodemographic data and community pharmacy details

Study population was 195 male and female CPs of which 55.4% (*n* = 108) were male and 44.6% (*n* = 87) were female. Most participants were in the age of ‘30 years and above’ (69.7%; *n* = 136). Eighty-six (44.1%) of the enrolled pharmacists were being in the in-charge position. Of the respondents, 172 (88.2%) were working in chain pharmacies and mostly (64.1%; *n* = 125) were located on the main streets. The vast majority of the participants were holding a postgraduate degree (65.1%; *n* = 127). Ninety-seven percent had access to internet in the pharmacy. The average time spent with patient by the pharmacists was 5.68 (SD ± 2.45) min. Further details are in Table 1.

**Table 1:** Pharmacist and pharmacy-related information (*n* = 195)

Variables		*n* (%)
Gender	Male Female	108 (55.4) 87 (44.6)
Age	Below 30 Above or equal 30	59 (30.3) 136 (69.7)
Country of original of the respondents	UAE South eastern Asia Arab country other than UAE Others	10 (5.1) 22 (11.3) 134 (68.7) 29 (14.9)
Attainment of postgraduate certificate	Yes No	68 (34.9) 127 (65.1)
Postgraduate qualification	No postgraduate Diploma Master PhD	127 (65.1) 18 (9.2) 48 (24.6) 2 (1.0)
Pharmacy location	Main street Shopping center Near to medical center Others	125 (64.1) 18 (9.2) 35 (17.9) 17 (8.7)
Respondents’ position in the pharmacy	Proprietor Pharmacists in charge Employee pharmacist Others	3 (1.5) 86 (44.1) 105 (53.8) 1 (0.5)
Pharmacy type	Chain Individual	172 (88.2) 23 (11.8)
Access to the internet	Yes No	190 (97.4) 5 (2.6)
Availability of drug information sources	Internet British national formulary MOH website Lexicomp Drug index Nothing Martindale	139 (71.3) 41 (21.0) 4 (2.6) 2 (1.0) 3 (1.5) 4 (2.1) 1 (0.5)

Variables	Mean ± SD
Years after graduation	11.75 ± 6.68
Years after obtaining postgraduate	6.39 ± 3.66
Practice years	10.09 ± 6.45
Average weekly working hours	49.47 ± 9.63
Average number of prescriptions dispensed per day	30.34 ± 10.94
Average number of patients served a day	38.89 ± 13.54
Number of pharmacists working with	2.97 ± 0.91
Number of non-pharmacist employees in pharmacies	1.06 ± 1.07
Average time spent with patient	5.68 ± 2.45

### Community pharmacists perception towards ECPs

Of the respondents, 71% (*n* = 140) ‘strongly agreed’ that every CP must provide ECPs and 39% (*n* = 76) ‘strongly agreed’ in supporting the concept of ECPS. Nearly one third (32.6%; *n* = 63) of the CPs ‘agreed’ that it is difficult to implement ECPS in UAE due to language barriers. Further details of the respondents’ perception towards ECPS are mentioned in Table 2.

**Table 2:** Community pharmacists’ perception toward provision of ECPS

Statement	Strongly agree, *n* (%)	Agree, *n* (%)	Neutral, *n* (%)	Disagree, *n* (%)	Strongly disagree, *n* (%)
1. Every CP must provide ECPS	140 (71.8)	37 (19)	15 (7.7)	3 (1. 5)	0
2. I fully support the concept of ECPS	76 (39)	86 (44.1)	27 (13.8)	6 (3.1)	0
3. ECPS is really the doctor’s role^a^	110 (54.5)	78 (40)	7 (3.6)	0	0
4. ECPS requires major up-skilling of clinical knowledge	104 (53.3)	67 (34.4)	24 (12.3)	0	0
5. Doctors and other health professionals will not support an ECPS role for pharmacists^a^	72 (36.9)	35 (17.9|)	34 (17.4)	36 (18.5)	18 (9.20
6. CPs alone cannot provide ECPS^a^	24 (12.3)	20 (10.3)	51 (26.2)	79 (40.5)	21 (10.8)
7. My pharmacy education never provided me with skills needed for ECPS^a^	16 (8.2)	34 (17.4)	29 (14.9)	71 (36.4)	45 (23.1)
8. I may lose my patients if I start providing ECPS to them^a^	0	5 (2.6)	38 (19.5)	76 (39)	76 (39)
9. ECPS is good to market pharmacy services	0	17 (8.7)	105 (53.8)	58 (29.7)	15 (7.70
10. ECPS can be used to generate extra revenue	0	10 (5.1)	103 (52.8)	68 (34.9)	14 (7.2)
11. ECPS is difficult to implement in UAE due to language barrier^a^	9 (4.6)	63 (32.6)	64 (32.8)	50 (25.6)	9 (4.6)

^a^Negative perception item

### Community pharmacists existing practice of ECPS

With regard to the current practice of CPs toward ECPS, their median responses ranged from 2 (rare) to 3 (occasionally). ECPS services with 2 (rare) as median score where related to the following: weight management 2 (2–3), diabetes patient monitoring 2 (2–3), BMI calculation 2 (1–3), counselling on oral contraceptives 2 (1–4), back pain management 2 (1–3), care to pregnant patients (nutrition, abdominal belts, etc.) 2 (1–3), and assessment of peripheral neuropathy 2 (1–3). ECPS services with 3 (occasionally) as median score where related to the following: smoking related 3 (2–3), BP measurement 3 (1–4), cholesterol measurement 3 (2–4), counselling on family planning 3 (2–5), dietary supplements 3 (1–4), and pregnancy testing 3 (3–4).

### Community pharmacists’ perceived barriers data in providing ECPS

In the current study, 26 statements were used to assess pharmacists perceived barriers towards provision of ECPS. These statements were categorized into five main domains of factors: attitudinal, skill-set, resource-related, system-related and patient-related. The major attitude-related barrier was the lack of incentive for CPs [median (IQR) score; 3 (2–3)], major barrier related to the lack of skill-set factors was ‘lack of documentation [median (IQR) score; 3 (2–3)], major resource-related barriers were ‘insufficient time’ and ‘motivation’, both having a median (IQR) scores of 3 (2–3). The major system-related barriers were ‘lack of reimbursement’ and ‘lack of patient demand’ both with median (IQR) scores of 3 (2–3). The details are presented in Table 3.

**Table 3:** Community pharmacists’ perceived barriers in providing ECPS

I find the following barriers in providing ECPS		Median (IQR score)
Attitudinal factors	My level of understanding of ECPS	1 (1–2)
	Other pharmacists’ attitudes towards ECPS	1 (1–2)
	Fear of change among pharmacists	2 (1–2)
	Lack of motivation among pharmacists	2 (1–3)
	Lack of confidence among pharmacists	2 (2–3)
	Lack of incentive for employee pharmacists	3 (2–3)
Skill-set factors (lack of advanced practice Skills)	Lack of therapeutics knowledge among pharmacists	2 (1–3)
	Lack of clinical problem-solving skills	1 (1–2)
	Lack of communication skills	1 (1–2)
	Lack of specific training	2 (1–3)
	Lack of documentation (processes/software)	3 (2–3)
	Lack of drug information resources (processes/access)	2 (1–3)
Resource-related constraints	Insufficient time	3 (2–3)
	Insufficient finances	2 (1–3)
	Appropriate physical space	2 (1–3)
	Motivated personnel (e.g., pharmacists, technicians)	3 (2–3)
	Appropriate management systems (e.g., workflow)	2 (2–3)
System-related constraints	Lack of reimbursement system	3 (2–3)
	Lack of patient demand	3 (2–3)
	Doctor/nurse resistance	2 (1–3)
	Lack of access to patient medical records	2 (1–3)
	Lack of data on value of ECPS	1 (1–2)
Patient related	Patients are not willing	2 (1–3)
	Patients are usually busy	1 (1–2)
	Non-willingness for payment	2 (1–2)
	Lack of private consultation room	2 (1–2)

### Willingness of the CPs in providing ECPS

The CPs’ willingness response toward ECPS ranged from 2 (partly) to 3 (completely). CPs responded as ‘completely’ willing to provide services such as blood pressure measurement, pregnancy testing and BMI measurement, etc. Details are provided in Table 4. Among the respondents, 38 (19.48%) responded to the open-ended question. Of them, 31 indicated that they are willing to do testing and laboratory work in the community pharmacies, while, seven mentioned that medication therapy counselling is an example of the ECPS they are willing to apply in their practice.

**Table 4:** Community pharmacist’s willingness to provide specific ECPS

Services	Median (IQR score)
Blood pressure measurement	3 (3–3)
Pregnancy testing	3 (3–3)
Blood sugar testing	3 (2–3)
Body mass index	3 (3–3)
Ear piercing	2 (2–3)
Body fat analysis	2 (1–3)

### Association between demographic variables and CPs perception, practice, barrier and willingness scores

There was a significate effect of the pharmacy location on the practice score of pharmacists (*p* = 0.008). While the access to the internet to community pharmacies has a significant effect on the barriers score (*p* = 0.000). In addition, there was a significant difference between the availability of the references and the perception score (*p* = 0.038), practice score (*p* = 0.000) and willingness score (*p* = 0.011). Details are illustrated in Table 5.

**Table 5:** Association between demographic variables and CPs perception, practice, barrier and willingness scores

Demographic characteristics of CPs	Intervals	Perception		Practice		Barriers		Willingness	
		Median (IQR) total score	P value	Median (IQR) total score	P value	Median (IQR) total score	P value	Median (IQR) total score	P value
Gender	Male Female	29 (28–31) 29 (28–31)	0.287^*^	32 (30–33) 32 (30–38)	0.944^*^	54 (51–58) 55 (50–61)	0.743^*^	15 (15–17) 15 (14–17)	0.802^*^
Age	Below 30 Above or equal 30	29 (28–31) 29 (28–31)	0.112^*^	31 (30–39) 32 (30–34)	0.902^*^	55 (50–61) 54 (50–58)	0.195^*^	16 (14–17) 15 (15–16)	0.240^*^
Nationality	Local South eastern Asia Arab Others	29 (27.75–29.5) 29.5 (28–31) 29 (28–31) 29 (28–32.5)	0.618^**^	39 (36–40) 31.5(30–39) 32 (30–38) 32 (30–32.5)	0.051^**^	52 (26–58) 52 (47.75–55.5) 55 (50–58) 55 (51–59.5)	0.325^**^	15.5 (14–18) 15 (14–17) 15 (15–17) 15 (14.5–16)	0.801^**^
Postgraduate certificate	Yes No	29 (28–31) 29 (28–32)	0.287^*^	32 (30–38) 32 (30–35)	0.183^*^	53.5 (43–57.75) 55 (51–59)	0.074^*^	15 (14–17) 15 (15–17)	0.746^*^
Postgraduate qualification	No postgraduate Diploma Master PhD	29 (28–32) 29 (28–30.25) 29 (28–31) –	0.760^**^	32 (30–35) 31 (30–33) 33 (30–39.75) –	0.222^**^	55 (51–59) 53 (46.75–57.25) 54.5 (41–58) –	0.321^**^	15 (15–17) 15.5 (14.75–17) 15 (14–17) 15 (15–15)	0.945^**^
Pharmacy location	Main street Shopping center Near to medical center Others	29 (28–31.5) 28.5 (26–31) 29 (28–30) 30 (26.5–32)	0.145^**^	32 (30–33) 32 (29–34.25) 38 (30–40) 30 (29–32)	0.008^**^	54 (50–58) 56 (52–66) 52 (26–58) 55 (50–57)	0.317^**^	15 (15–17) 16 (14.75–17) 15 (14–18) 15 (14–15)	0.249^**^
Your position in the pharmacy	Proprietor Pharmacists in charge Employee pharmacist Others	– 29 (28–31) 29 (28–31) 27 (27–27)	0.452^**^	– 32 (30–38) 31 (30–34) 31 (31–31)	0.286^**^	– 53.5 (50–58) 55 (50.5–60) 55 (55–55)	0.599^**^	– 15 (15–17) 15 (14–17) 16 (16–16)	0.881^**^
Pharmacy type	Chain Individual	29 (28–31) 29 (28–31)	0.617^*^	32 (30–38) 33 (30–39)	0.252^*^	55 (50–58) 52 (50–57)	0.633^*^	15 (15–17) 15 (12–17)	0.189^*^
Access to the internet	Yes No	29 (28–31) 29 (26.5–35)	0.779^*^	32 (30–38) 30 (29–43)	0.984^*^	54 (50–58) 85 (75.5–94.5)	0.000^*^	15 (15–17) 11 (11–14.5)	0.010^*^
References available	Internet BNF MOH website Lexicomp Drug index Nothing Martindale	29 (28–31) 29 (28–32) 31 (26.5–31.5) – – 32 (29–35) 27 (27–27)	0.038^**^	32 (30–33) 33 (30–39) 32 (28–33) 40 (40–40) – 42.5 (35–43) 29 (29–29)	0.000^**^	55 (50–58) 52 (50.5–57) 54 (51–57) – – 55.5 (26–85) 94 (94–94)	0.205^**^	15 (15–17) 16 (15–18) 15 (14.5–15) – – 14.5 (11–18) 11 (11–11)	0.011^**^

^*^Mann–Whitney U test; ^**^Kruskal–Wallis H test; Bold indicate statistically significant values

### Post hoc analysis

For those variables with statistical significance, Dunn post hoc analysis showed pharmacies located as ‘others’ and ‘near to medical center’ were significantly different from ‘other pharmacy locations’ that found to affect the practice score (0.007). Also, ‘drug index-no other resources’ (0.012) and ‘internet-no other resources’ (0.28) were significantly different from ‘other references’ that found to be significantly affecting the practice score, and ‘drug index-BNF’ were significantly different effecting the willingness score compared to other sub-variables (0.047).

## Discussion

The current study is considered to be the first its kind in the UAE that evaluated the perception, practice, perceived barriers and willingness of CPs toward the provision of ECPS. The findings showed a positive response from CPs and laid foundation on strengthening community pharmacy services in the country. Around the world, there are continued efforts offered by several pharmacy professional organizations to boost the provision of high level patient-oriented care in the community pharmacies [35, 36]. Establishing ECPS is one of the best strategies for the progress of community pharmacy practice [37]. However, despite widespread support, many obstacles have hindered the universal practice of ECPS [31, 38, 39].

Results showed CPs to have a positive perception towards provision of ECPS which is anticipated and similar to reported from countries other than the UAE [40]. Naturally, offering ECPS can help CPs in increasing their income and a professional satisfaction. A detail analysis of CPs practices showed they, on a routine basis, provided services which can be categorized as ECPS though this research did not assess the quality of these services or patient satisfaction to validate these services. These existing practices provide an opportunity for widening and strengthening the services with proper trainings and resources.

What was evident from the present study is that ‘level of understanding ECPS’, ‘lack of data on the value of ECPS’, and ‘lack of clinical problem-solving skills’, were considered as barriers to the practice of ECPS. The findings of this study do not differ much from studies conducted in other countries assessing the barriers of implementing pharmaceutical care [31, 33, 41, 42]. This study finding reflects that CPs are not sufficiently prepared with the required qualifications to support and enable them gain experience and confidence to practice extended services in their pharmacies. Definitely this problem could be resolved by the health authorities in the UAE through foundation of continuous educational seminars and conferences for CPs to improve their qualifications. Indeed, CPs must have expertise and skills that go beyond the traditional functions of community pharmacy [42, 43]. A competent and well-trained pharmacist would be able to provide more successful health services and would definitely have a beneficial influence on health concerns [44].

Also, in the current study, it was concluded that both gender considered the ECPS in an equal manner. Similar findings have been reported in Nigeria [33], but unlike findings of other study conducted in New Zealand [45]. This observation reflects the brilliant success of UAE government in achieving the gender education equality, although, many obstacles are standing in the way of women that fully exercising their right to participate in, complete and benefit from education in many countries.

Furthermore, our study revealed that modern generation pharmacists (aged below 30) do not have much difference than the previous generation pharmacist (aged above or equal to 30) in term of ECPS. These findings advocate the need of a coordinated and collaborative working between ministry of higher education and pharmacy colleges in UAE to improve the pharmacy curriculum through exposure to the principles and practice of ECPS as part of early pharmacy education for successful implementation of ECPS within the community pharmacies. The improvement in pharmacy curriculum has to meet the new changes in the pharmacy profession and the patients` demands and desires. Moreover, the CPs in UAE are educated outside UAE and probably with countries with little of professional community pharmacy services, suggesting needs for skill enhancement trainings.

The current study reported no significant differences in the responses recorded by pharmacists holding postgraduate certificate and others, unlike the studies conducted in Nigeria and Scotland [13, 33]. This is probably because they have not been adequately trained and are therefore less competent and confident in taking on new roles. This finding requires serious attention and a continued effort to try to make a difference by providing appropriate and suitable training and opportunities for CPs that meet their needs to improve the future pharmacy practice.

It was found in this current study that the presence of internet access at pharmacy significantly affect the willingness to practice ECPS, which is compatible with results concluded in previous studies conducted in Scotland, India, England [46–48]. Access to internet can ease the information transfer, which will give the CPs the opportunity to learn, even if they do not have good education [49]. Thus, CPs will become more knowledgeable about new pharmacy practice approaches like ECPS. This will encourage them to practice patient-oriented approaches rather than only dispensing, which is at the heart of ECPS concept. These figures should convince the health authorities policy-makers of the significance of the internet availability in the pharmacies. Therefore, it is suggested to make the internet access mandatory in all pharmacies in UAE.

The results of the present study have indicated that there were significant differences between the perception, practice and willingness score and the availability of the resources in the pharmacy. This finding should be taken with optimism in contemplating widespread introduction of ECPS. These observations reflect the possibility of the health authorities to make changes for the development of ECPS among CP by insuring the free access to different online resources in pharmacies.

Based on the reporting of respondents in the present study, pharmacy located near medical centers appeared to be more likely to practice the ECPS. One notable illustration is that these pharmacies are more accessible to chronically ill patients (who are usually willing to accept ECPS practice). So, these community pharmacies are considered as an ideal environment to fulfill the requirements and the desire of these patients, by providing high-standard extended services.

The major limitation of this study is that the findings were restricted to only CPs working in three emirates in UAE, Sharjah, Ajman and Umm Al Quwain. The outcomes would have been more significant if the study was conducted in all UAE cities. In addition, sample size completion was affected due to the COVID-19 lockdown, even though the number of participating pharmacists 195 was adequate to obtain a fair response rate. This study did not assess the clinical skills of CPs in providing ECPS and hence it is unclear whether the responses of the CPs were influenced by their lack of confidence in performing such services. Since this research is survey based, a Dunning–Kruger effect cannot be ignored.

## Conclusions

CPs had a positive perception on providing ECPS, willing to provide ECPS and were practicing ECPS within their purview of available resources and facilities. They felt lack of time and patient demand as the major barriers and most barriers can be overcome by education, training and remuneration to pharmacists. A proper utilization of CPs in providing ECPS can complement the healthcare system in UAE and can potentially save time and money for the patients. The results have emphasized the critical need for interventions to promote ECPS, to resolve the obstacles defined by the study, maintain the positive perceptions of the CPs and to improve the knowledge and practice toward ECPS. Policy-makers should adopt diverse methods to enhance the involvement of CPs in ECPS.

## Supplementary Information

**Additional file 1:** Study questionnaire.

## Data Availability

The data for the research are available from the corresponding author with a reasonable request.

## Abbreviations

CP: Community pharmacist
CPs: Community pharmacists
ECPS: Extended community pharmacy services
HCPs: Healthcare providers
UAE: United Arab Emirates
UK: United Kingdom
US: United States
HAAD: Health Authority of Abu Dhabi
DHA: Dubai Health Authority
