# Supplementary material for: Opportunities for extended community pharmacy services in United Arab Emirates: perception, practice, perceived barriers and willingness among community pharmacists
Source: J Pharm Policy Pract. 2022 Mar 23;15:24. doi: 10.1186/s40545-022-00418-y (PMC8941297; doi:10.1186/s40545-022-00418-y)
Supplement: Supplementary file 1 — Additional file 1: Study questionnaire. [file 40545_2022_418_MOESM1_ESM.docx]

|  |  |
| --- | --- |

Title: A survey exploring the perception, practices, perceived barriers and willingness of community pharmacists in UAE towards provision of Extended community pharmacy services (ECPS)

Please take a few minutes to answer the following statements below.

**Instructions**

-Please read each question carefully before answering

- Choose the answer that best describes what you believe and feel to be correct.

**Pharmacist and pharmacy related information (Part I)**

1. **Gender:**
2. Male
3. Female
4. **Age (in 2020):…………**

**3. Nationality of the pharmacist:**

- 1. Local (UAE)
  2. South eastern Asia
  3. Arab
  4. Others --------------
     1. **Year of graduation:……….**
     2. **How long have you been practicing as a community pharmacist (years)?..............**
     3. **Do you have any postgraduate certificate?**

1. Yes
2. No

**If “yes”: please specify**

Qualification --------------------------

Year obtained -------------------------

- - 1. **The location of your pharmacy?**

1. Main street
2. Shopping Centre
3. Near to medical Centre
4. Others --------------
   - 1. **Which of the followings best describe your position in the pharmacy?**

*(you can choose more than one option if applicable)*

1. Proprietor (owner) or partner proprietor
2. Pharmacist in-charge
3. Employee pharmacist

Others --------------------

- - 1. **Average hour of weekly hours worked:………**
    2. **Average number of prescriptions dispensed per day?.................**
    3. **Average number of patients served day approx.: ……….**

- - 1. **The type of pharmacy you currently working in:**

1. Chain
2. Individual
   - 1. **Number of pharmacists you work with:**

**---------**

- - 1. **Number of employees (other than pharmacist): -----------**
    2. **Mention any references available in your pharmacy:**

**………………………………………………….**

**………………………………………………….**

- - 1. **Average time spent with patient ……………..**
    2. **Access to the internet is available in the pharmacy:**

1. Yes
2. No

**Community pharmacist’s perception towards ECPS (Part II)**

| Statement | Strongly agree | Agree | Neutral | Disagree | Strongly disagree |
| --- | --- | --- | --- | --- | --- |
| Every community pharmacist must provide ECPS |  |  |  |  |  |
| I fully support the concept of ECPS |  |  |  |  |  |
| ECPS is really the Doctor’s role |  |  |  |  |  |
| ECPS requires major up-skilling of clinical knowledge |  |  |  |  |  |
| Doctors and other health professionals will not support an ECPS role for pharmacists |  |  |  |  |  |
| Community pharmacists along cannot provide ECPS |  |  |  |  |  |
| My pharmacy education never provided me with skills needed for ECPS |  |  |  |  |  |
| I may lose my patients if I start providing ECPS to them |  |  |  |  |  |
| ECPS is good to market pharmacy services |  |  |  |  |  |
| ECPS can be used to generate extra revenue |  |  |  |  |  |
| ECPS is difficult to implement in USE due to language barrier |  |  |  |  |  |

**Community pharmacist’s practices towards ECPS (Part III)**

**What are the community pharmacists` practice toward ECPS?**

| Services | Never | Rare | Occasionally | Often | Always |
| --- | --- | --- | --- | --- | --- |
| Weight management |  |  |  |  |  |
| Smoking related |  |  |  |  |  |
| Diabetes patient monitoring |  |  |  |  |  |
| BMI calculation |  |  |  |  |  |
| BP measurement |  |  |  |  |  |
| Cholesterol measurement |  |  |  |  |  |
| Services | Never | Rare | Occasionally | Often | Always |
| Counseling on oral contraceptives |  |  |  |  |  |
| Counseling on family planning |  |  |  |  |  |
| Dietary supplements |  |  |  |  |  |
| Pregnancy testing |  |  |  |  |  |
| Back pain management |  |  |  |  |  |
| Care to pregnant patients (nutrition, abdominal belts, etc) |  |  |  |  |  |
| Assessment of peripheral neuropathy |  |  |  |  |  |

**Community pharmacist perceived barriers towards provision of ECPS (Part IV)**

| I find the following barriers when providing ECPS: | | Strongly Agree | Agree | Neutral | Disagree | Strongly Disagree |
| --- | --- | --- | --- | --- | --- | --- |
| **Attitudinal Factors** | My level of understanding of ECPS |  |  |  |  |  |
|  | Other pharmacists’ attitudes towards ECPS |  |  |  |  |  |
|  | Fear of change among pharmacists |  |  |  |  |  |
|  | Lack of motivation among pharmacists |  |  |  |  |  |
|  | Lack of confidence among pharmacists |  |  |  |  |  |
|  | Lack of incentive for employee pharmacists |  |  |  |  |  |
| **Skill-Set Factors (Lack of Advanced practice Skills)** | Lack of therapeutics knowledge among pharmacists |  |  |  |  |  |
|  | Lack of clinical problem-solving skills |  |  |  |  |  |
|  | Lack of communication skills |  |  |  |  |  |
|  | Lack of specific training |  |  |  |  |  |
|  | Lack of documentation (processes/software) |  |  |  |  |  |
|  | Lack of drug information resources (processes/access) |  |  |  |  |  |
| **Resource Related Constraints** | Insufficient time |  |  |  |  |  |
|  | Insufficient finances |  |  |  |  |  |
|  | Appropriate physical space |  |  |  |  |  |
|  | Motivated personnel (e.g. pharmacists, technicians) |  |  |  |  |  |
|  | Appropriate management systems (e.g. workflow) |  |  |  |  |  |
| **System-Related Constraints** | Lack of reimbursement system |  |  |  |  |  |
|  | Lack of patient demand |  |  |  |  |  |
|  | Doctor/nurse resistance |  |  |  |  |  |
|  | Lack of access to patient medical records |  |  |  |  |  |
|  | Lack of data on value of ECPS |  |  |  |  |  |
| **Patient**  **Related** | Patients are not willing |  |  |  |  |  |
|  | Patients are usually busy |  |  |  |  |  |
|  | Non-willingness for payment |  |  |  |  |  |
|  | Lack of private consultation room |  |  |  |  |  |

**Community pharmacist’s willingness to provide ECPS (Part V)**

I am competent to perform the following services if I am provided with all necessary support (managerial, regulatory and motivational):

| Services | Never | Partly | Completely |
| --- | --- | --- | --- |
| Blood pressure measurement |  |  |  |
| Pregnancy testing |  |  |  |
| Blood sugar testing |  |  |  |
| BMI |  |  |  |
| Ear piercing |  |  |  |
| Body fat analysis |  |  |  |
| Any other (please mention……….. |  |  |  |
|  |  |  |  |

**THANK YOU FOR YOUR VALUABLE TIME**
